# ZAR1: Guardian of plant kinases

**DOI:** 10.3389/fpls.2022.981684

**Published:** 2022-09-23

**Authors:** Clare Breit-McNally, Bradley Laflamme, Racquel A. Singh, Darrell Desveaux, David S. Guttman

**Affiliations:** ^1^ Department of Cell and Systems Biology, University of Toronto, Toronto, ON, Canada; ^2^ Centre for the Analysis of Genome Evolution & Function, University of Toronto, Toronto, ON, Canada

**Keywords:** ZAR1, ZRK, PBL, kinases, pseudokinases, network, effector-triggered immunity, arabidopsis

## Abstract

A key facet of innate immunity in plants entails the recognition of pathogen “effector” virulence proteins by host Nucleotide-Binding Leucine-Rich Repeat Receptors (NLRs). Among characterized NLRs, the broadly conserved ZAR1 NLR is particularly remarkable due to its capacity to recognize at least six distinct families of effectors from at least two bacterial genera. This expanded recognition spectrum is conferred through interactions between ZAR1 and a dynamic network of two families of Receptor-Like Cytoplasmic Kinases (RLCKs): ZED1-Related Kinases (ZRKs) and PBS1-Like Kinases (PBLs). In this review, we survey the history of functional studies on ZAR1, with an emphasis on how the ZAR1-RLCK network functions to trap diverse effectors. We discuss 1) the dynamics of the ZAR1-associated RLCK network; 2) the specificity between ZRKs and PBLs; and 3) the specificity between effectors and the RLCK network. We posit that the shared protein fold of kinases and the switch-like properties of their interactions make them ideal effector sensors, enabling ZAR1 to act as a broad spectrum guardian of host kinases.

## Introduction

As sessile organisms, plant species are required to perceive and respond to environmental stimuli encompassing a wide range of abiotic and biotic interactions. This perception is mediated through various receptor classes, which function both extra- and intracellularly to perceive a broad range of potential perturbations (Hohmann et al., 2017; Wang and Chai, 2020). Recently, considerable attention has been paid toward the capacity of plant receptors to form complex networks through their interactions with one another. In *Arabidopsis thaliana*, the extracellular domains of leucine-rich repeat receptor kinases form a complex and multi-layered network through physical interactions to regulate their roles in a wide range of biological functions (Smakowska-Luzan et al., 2018). This receptor network provides *A. thaliana* with the regulatory complexity necessary to integrate information about its external environment into a manageably sized repertoire of cell-surface receptors. Intracellularly, Nucleotide-Binding Leucine-Rich Repeat Receptors (NLRs) also form extensive networks, both with other NLRs and distinct receptor classes, to mediate immunity against a broad range of pathogens (Wu et al., 2018; Adachi et al., 2019). NLRs govern the recognition of intracellular pathogen virulence proteins (often termed “effectors”) to activate Effector-Triggered Immunity (ETI) (Jones and Dangl, 2006). Effectors are secreted into host cells by pathogens spanning several kingdoms of life, and the extraordinary number and diversity of effector proteins necessitates a strong NLR surveillance network (Ngou et al., 2022). NLR networks are composed of ‘sensor’ NLRs (sNLR) that directly or indirectly perceive pathogenic effectors and interact with a handful of ‘helper’ NLRs (hNLR), which are responsible for immune signal dissemination through their acting as calcium-permeable channels (Bonardi et al., 2011; Wu et al., 2017; Saile et al., 2020; Jacob et al., 2021). As such, NLR networks can be divided into components with specific roles in pathogen recognition (sensors) and components that function more generically in signaling (helpers).

**Figure 1:**
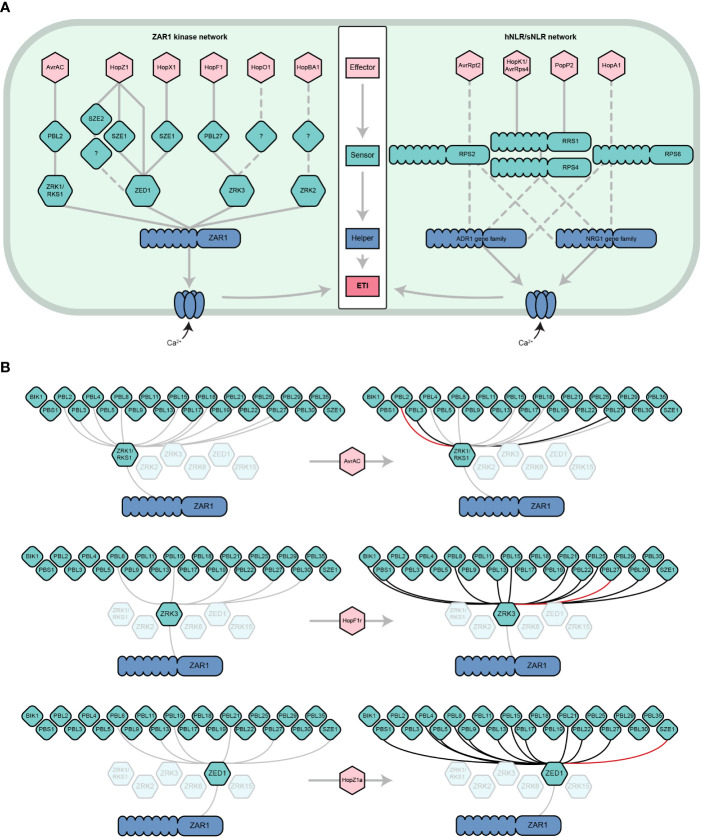
The dynamic ZAR1/kinase immune signaling network. **(A)** Parallels between the ZAR1/kinase (left) and hNLR/sNLR (right) immune signaling networks. ZAR1 and hNLRs play similar roles in *A. thaliana* ETI. Six effector families are indirectly recognized by ZAR1 through its interactions with ZRK (green hexagons) and PBL (diamonds) kinase sensors (Wang et al., 2015; Seto et al., 2017; Liu et al., 2019; Martel et al., 2020; Seto et al., 2021). Analogously, several distinct effector families are recognized by sNLRs, which go on to activate NRG1 and/or ADR1 hNLRs (only a subset are represented) (Bonardi et al., 2011; Dong et al., 2016; Castel et al., 2019; Wu et al., 2019). Like the ZAR1 resistosome, hNLRs oligomerize and localize to the cell membrane to form calcium-permeable channels. Solid lines represent physical interactions whereas dashed lines represent signaling dependencies, which may include physical interactions that have yet to be demonstrated. Other ETI components of the sNLR/hNLR network (e.g., EDS1, NDR1) have been omitted for simplicity. This panel is adapted from Martel et al., 2020 and Jubic et al., 2019. **(B)** ZRK-PBL interactions induced by the effectors AvrAC, HopF1r, and HopZ1a according to the results of Bastedo et al., 2019; Seto et al., 2021, and Martel et al., 2020. Grey lines represent physical interactions that occur in the absence of an effector, black lines represent physical interactions that are induced or strengthened by the presence of an effector, and red lines represent the ZRK-PBL interaction required for the ETI response to the given effector in *A. thaliana*. Shown are the ZRKs that have been demonstrated to interact with ZAR1 (Wang et al., 2015) and the PBLs that have been demonstrated to interact with ZRKs (Bastedo et al., 2019; Seto et al., 2021). For more information about effector nomenclature, refer to (Lindeberg et al., 2005).

The ZAR1 NLR is widespread throughout flowering plants and presents a variation on the canonical NLR network. Like hNLRs, ZAR1 forms a calcium-permeable channel upon activation, however it is activated by a dynamic network of ‘sensor’ kinases rather than sNLRs (**Figure 1A**) (Wang et al., 2019a; Wang et al., 2019b; Adachi et al., 2021; Bi et al., 2021). Here, we review the history of functional studies on ZAR1, with a particular emphasis on its remarkable network of interactions with Receptor-Like Cytoplasmic Kinases (RLCKs) that confer a broad effector recognition profile. We argue that through its elaborate set of interactions with switch-like kinases, ZAR1 can effectively monitor for perturbations to a broad range of kinases that are frequently targeted by pathogenic effectors.

## ZAR-once upon a time: a brief history of studies on ZAR1

ZAR1 (HOPZ-ACTIVATED RESISTANCE1) was first characterized for its genetic requirement in the recognition of the *Pseudomonas syringae* acetyltransferase effector HopZ1a (Ma et al., 2006; Lewis et al., 2010). HopZ1a recognition through ZAR1 did not require known ETI signaling genes, suggesting that novel ETI components would be involved in ZAR1-mediated immunity (Lewis et al., 2010; Macho et al., 2010). In support of this, a later forward genetic screen identified an RLCK XII-2 pseudokinase *zed1* (*hopZ-ETI-deficient1*) mutant as also being deficient in HopZ1a ETI (Lewis et al., 2013). HopZ1a could acetylate ZED1 and mimicking these acetylation events was subsequently shown to be sufficient to activate ZAR1 (Lewis et al., 2013; Bastedo et al., 2019). Since ZED1 is a pseudokinase and *zed1* mutants did not display altered basal immune responses, it was hypothesized that ZED1 acts as a decoy that mimics kinase virulence targets of HopZ1a. In support of this hypothesis, HopZ1a has recently been shown to also target the kinase MKK7 (Mitogen-Activated Protein Kinase (MAPK) Kinase7) to suppress immunity (Rufián et al., 2021).

ZED1 is in a genomic cluster alongside other RLCK XII-2 family members (ZED1-Related Kinases, or ZRKs) in *A. thaliana* (Lewis et al., 2013). Members of this subfamily are considered atypical kinases because they lack at least one conserved kinase motif and most appear to be pseudokinases that lack kinase activity (Lewis et al., 2013; Roux et al., 2014). Several subsequent studies have implicated other ZRKs in ZAR1-mediated effector recognition. Following the identification of ZAR1, five additional effectors were identified to trigger ZAR1-mediated ETI in *A. thaliana* (Wang et al., 2015; Seto et al., 2017; Laflamme et al., 2020), all of which also require a specific ZRK for recognition. ZRK1 (also known as RESISTANCE RELATED KINASE1, RKS1) was first associated with resistance to *Xanthomonas campestris* in *A. thaliana* using genome-wide association mapping and was subsequently shown to be required for recognition of the *X. campestris* effector AvrAC (Huard-Chauveau et al., 2013). ZRK3 is required for ZAR1-mediated recognition of the *P. syringae* effector HopF1r (formerly HopF2a) (Seto et al., 2017) and ZED1, ZRK3 and ZRK2 are required for the recognition of HopX1i, HopO1c and HopBA1a, respectively (Martel et al., 2020). Overall, these studies collectively highlighted two remarkable aspects of ZAR1-mediated ETI: (1) that ZAR1 displays remarkable immunodiversity, recognizing at least six distinct effector families; and (2) that its immunodiversity is conferred by ZRK family members.

Studies on the recognition of the *X. campestris* effector AvrAC provided crucial mechanistic insights into ZAR1 activation, culminating in the first structure of a plant resistosome (Wang et al., 2019a; Wang et al., 2019b). AvrAC uridylylates members of the RLCK VII kinase family (a.k.a. PBS1-like or PBL) including Botrytis Induced Kinase 1 (BIK1), which plays an important role in immune signaling, to suppress basal immunity (Feng et al., 2012). In some *A. thaliana* ecotypes, AvrAC was found to induce an ETI response that was dependent on the RLCK VII family member PBL2 (Xu et al., 2008; Guy et al., 2013). The subsequent discovery that AvrAC ETI also required ZAR1 and ZRK1/RKS1 provided the first link between ZAR1 and the RLCK VII kinase family and provided the foundation for our current understanding of ZAR1 activation (Wang et al., 2015). Unlike the direct acetylation of the RLCK XII-2 ZED1 by HopZ1a, AvrAC uridylylates PBL2, which promotes the interaction of PBL2 with a preformed complex of ZRK1/RKS1 and ZAR1 (Wang et al., 2015). It is this effector-induced interaction between the RLCK VII kinase PBL2 and the RLCK XII-2 pseudokinase ZRK1/RKS1 that leads to ZAR1 activation (Wang et al., 2015). The mechanism of ZAR1 activation was revolutionized by the structures of the ZAR1-ZRK1/RKS1-PBL2^UMP^ complex (Wang et al., 2019a; Wang et al., 2019b; Bi et al., 2021). Uridylylated PBL2 acts as a nucleotide exchange factor that promotes ZAR1 ADP to ATP exchange, which activates ZAR1 (Wang et al., 2019b). Activated ZAR1 then oligomerizes and forms a wheel-like pentameric structure termed a “resistosome”, which acts as a calcium-permeable cation channel that activates ETI (Wang et al., 2019a; Wang et al., 2019b; Hu et al., 2020; Bi et al., 2021). Focused reviews on the ZAR1 resistosome and subsequently defined resistosome structures for other plant NLRs can be found elsewhere (Burdett et al., 2019; Mermigka and Sarris, 2019; Bi and Zhou, 2021). Since HopZ1a creates a ZAR1-ZED1 complex of similar molecular weight to the ZAR1-ZRK1/RKS1-PBL2 resistosome in *A. thaliana* protoplasts (Hu et al., 2020), it is likely that similar ZAR1 resistosome structures are formed by distinct effector perturbations to the ZRK/PBL kinase network.

## The ZAR1-RLCK network

While the recognition spectrum of ZAR1 is broad, the ZAR1-associated ZRK/PBL network displays effector specificity. As outlined above, ZRK requirements have been identified for all known ZAR1-mediated ETI responses in *A. thaliana*; ZED1 is required for HopZ1a and HopX1i ETI (Lewis et al., 2013; Martel et al., 2020), ZRK3 is required for HopO1c and HopF1r ETI (Seto et al., 2017; Martel et al., 2020), ZRK1/RKS1 is required for AvrAC ETI (Wang et al., 2015), and ZRK2 is required for HopBA1a ETI (Martel et al., 2020). In addition to ZRKs, PBLs have also been shown to be required for ZAR1-mediated ETI responses beyond AvrAC: PBL27 is required for HopF1r ETI (Seto et al., 2021), SZE1 is required for HopX1i (Martel et al., 2020), and both SZE1 and SZE2 contribute to HopZ1a ETI (Liu et al., 2019). SZE1/2 are divergent members of the RLCK VII family (Liu et al., 2019). Overall, although six effector families can activate ZAR1 ETI, each requires a specific ZRK/PBL combination for its recognition.

Activation of ZAR1 through its associated RLCK network can occur through effector-mediated modifications of either ZRKs or PBLs. AvrAC uridylylates PBL2, which promotes its interaction with a preformed ZAR1-ZRK1/RKS1 complex (Wang et al., 2015). Similarly, HopF1r ADP-ribosylates PBL27 and promotes its interaction with ZRK3 (Seto et al., 2017). An AvrAC-type recognition mechanism is proposed for HopF1r recognition, whereby PBL27 ribosylation leads to its association with ZRK3, which has been shown to be in a preformed ZAR1 complex (Wang et al., 2015; Seto et al., 2017). In support of this mechanism, the small molecule Zaractin that promotes the interaction between PBL27 and ZRK3, also activates ZAR1-dependent immunity in *A. thaliana* (Seto et al., 2021). On the other hand, HopZ1a acetylates ZED1, modifying its interactions with several PBLs (Lewis et al., 2013; Bastedo et al., 2019; Liu et al., 2019). Perturbations of ZED1, including HopZ1a acetyl-mimics, are sufficient to activate ZAR1, demonstrating that effectors can also directly modify ZRKs to modulate their interactions with PBLs and activate ZAR1. SZE1 and ZED1 occur in a preformed complex (Liu et al., 2019), and their interaction is enhanced by HopX1i, although the mechanism of HopX1i-induced SZE1/ZED1 perturbation has yet to be uncovered (Martel et al., 2020). PBLs are also hypothesized to be involved in ETI responses to HopO1c and HopBA1a, but these have yet to be identified. Overall, we hypothesize that ZRK nucleotide exchange factor function can be activated by interactions with various PBLs, and that this exchange can be promoted by perturbations to either kinase family. The ZRK may be in a preformed ZAR1 complex as observed for ZAR1-ZRK1/RKS1-PBL2, or alternatively, preformed ZRK/PBL dimers may bind to ZAR1, as suggested for HopZ1a ZED1/PBL interactions (Bastedo et al., 2019).

Although specific ZRK/PBL interactions can activate ZAR1 immunity, recent studies have revealed that ZRKs can associate with multiple PBLs beyond those required for ETI (Bastedo et al., 2019; Seto et al., 2021). A yeast-3-hybrid screen to investigate interactions between ZED1 and 46 A*. thaliana* PBLs in the presence and absence of HopZ1a (**Figure 1B**) found that HopZ1a promotes or enhances the interaction of ZED1 with 11 PBLs (PBL21, PBL22, PBL5, PBS1, PBL27, PBL17, PBL8, PBL4, PBL9, PBL15 and PBL13) (**Figure 1B**) (Bastedo et al., 2019). The closely related effector allele HopZ1b, which does not trigger ETI in *A. thaliana*, only induces a subset of these PBL-ZED1 interactions, indicating that most are specific to HopZ1a (Bastedo et al., 2019). The same approach was used to investigate interactions between ZRK1/RKS1 and PBLs in the presence of AvrAC. In this screen, AvrAC enhanced the interactions of ZRK1/RKS1 with PBL2, PBL3, and PBL29 (**Figure 1B**), despite only PBL2 being required for AvrAC ETI. In a similar analysis of ZRK3-PBL interactions, it was observed that HopF1r promoted stronger interactions between ZRK3 and 11 PBLs (PBL15, PBL27, PBL21, PBL30, PBL8, PBL25, PBL22, BIK1, PBS1, PBL13 and SZE1), but only PBL27 was required for HopF1r ETI (**Figure 1B**) (Seto et al., 2021). Although most of these effector-induced ZRK-PBL interactions have no known role in ETI, they may function under certain genetic or environmental contexts, or in some cases multiple ZRK-PBL interactions may have an additive effect on the ETI outcome as observed for HopZ1a (Liu et al., 2019). Further, these effector-PBL interactions may represent virulence targets, such as the targeting of BIK1 by HopF1r (Seto et al., 2021). Overall, these studies emphasize that ZRKs and PBLs form an expansive and dynamic network that is perturbed by pathogenic effectors, resulting in subsets of interactions that are detected by ZAR1. It is likely that more ZRKs and PBLs are involved in ZAR1-mediated immunity since there are at least 48 PBLs (Bastedo et al., 2019; Liu et al., 2019) and 13 ZRKs (Lewis et al., 2013) in *A. thaliana*, with four additional ZRKs in the same genomic cluster as ZED1, ZRK2, ZRK3 and ZRK1/RKS1, including ZRK6 which has been shown to interact with ZAR1 (Lewis et al., 2013; Wang et al., 2015).

## Sound the alarm: kinases as highly effective sensors of effectors

The capacity of kinases to both sense pathogen activity and transduce immune responses makes these proteins key to the overall immune architecture of plants. Immune signaling relies on RLCKs to activate several downstream responses including MAPK cascades, calcium flux, and the production of reactive oxygen species during Pattern-Triggered Immunity (PTI) (Yuan et al., 2021). However, this significance also makes kinases prime virulence targets of pathogen effectors to suppress plant immunity (Khan et al., 2018). Indeed, kinases are the most common characterized targets of bacterial effectors and are prominent targets of oomycete and fungal effectors as well (Khan et al., 2018). For instance, multiple effectors target MAPKs such as MPK4 and MPK6, PBLs such as RPM1-induced protein kinase (RIPK) and BIK1, and Receptor-Like Kinases (RLKs) such as FLS2 and BAK1 (Buttner, 2016; Khan et al., 2018). Fortunately, the ability of kinases to operate as ‘molecular switches’ (undergoing a conformational change that transitions the kinase from inactive (off) to active (on) states in response to external stimuli) makes them ideal broad spectrum effector sensors (Taylor et al., 2021). While the majority of ZRKs are pseudokinases that lack catalytic activity, PBLs possess functional kinase domains (Roux et al., 2014). It is unclear whether effector-induced effector-induced post-translational modifications (PTMs) alter PBL kinase activity, however effector-modified PBLs can activate the nucleotide exchange factor activity of ZRKs (Wang et al., 2019b). As described above, the protein kinase fold of ZRKs and PBLs also plays a crucial non-catalytic role in mediating protein-protein interactions that are modulated by effector-induced effector-induces PTMs (Mace and Murphy, 2021). Interestingly, the switch-like mechanism that makes kinases efficient PTM sensors may not be effective at detecting effector proteases, since cleavage of PBS1 by HopAR1 activates RPS5 rather than ZAR1 (Shao et al., 2003; Ade et al., 2007; Deyoung et al., 2012). As such, although ZAR1 is an effective guardian of effector-induced kinase PTMs (e.g., acetylation, uridylylation, ADP-ribosylation), recognition of kinase cleavage events induced by effector proteases requires a distinct NLR, and likely a distinct mechanism. Distinct recognition mechanisms for PTMs versus cleavage may also apply to the intrinsically disordered protein sensor RPM1 INTERACTING PROTEIN 4 (RIN4), which functions as an ETI signaling hub targeted by multiple effectors, whereby phosphorylation or acetylation of RIN4 are recognized by RPM1 but cleavage is recognized by RPS2 (Mackey et al., 2002; Axtell and Staskawicz, 2003; Mackey et al., 2003; Liu et al., 2011; Choi et al., 2021).

Although some sensor kinases may represent bona fide effector virulence targets, those that have been identified to date appear to be decoy mimics of virulence targets with ETI-specific roles as effector sensors (Khan et al., 2016). ZRK family members are primarily pseudokinases with ETI-specific roles, since deletion of the ZRK cluster in *A. thaliana* has no apparent effect on PTI or plant development (Lewis et al., 2013; Seto et al., 2020). However, ZRKs may play a role in temperature-regulated immunity (Wang et al., 2019c). ZED1 appears to be a decoy substrate of HopZ1a, which also targets the immunity-related kinase MKK7 (Rufián et al., 2021). In addition to PBL2, AvrAC uridylylates the immunity-related PBL kinases BIK1 (a paralog of PBL2) and RIPK (a PBL that phosphorylates RIN4, leading to RPM1-mediated ETI) to dampen PTI and ETI immune responses, respectively (Veronese et al., 2006; Feng et al., 2012). However, PBL2 plays only a small role in PTI signaling (Zhang et al., 2010; Wang et al., 2015). The conserved kinase protein structure likely results in inadvertent effector modifications to ‘sensors’ in addition to virulence targets, such as the catalytic- and activation-loop modifications observed for HopZ1a and AvrAC, respectively. Interestingly, HopZ1a acetylates different catalytic loop residues in the sensor ZED1 and virulence target MKK7 (Lewis et al., 2013; Rufián et al., 2021), while AvrAC uridylylates conserved activation loop residues in BIK1, RIPK, and PBL2 (Wang et al., 2015). Overall, ZRK/PBL sensors act as decoy mimics of kinase virulence targets that can include kinases outside the ZRK/PBL RLCK families, emphasizing the effectiveness of using the structurally conserved kinase fold as an effector sensor.

## Conservation of the ZAR1-ZRK-PBL network in other plant species

Support for the hypothesis that the ZAR1-ZRK-PBL network is conserved in other plant species comes from evolutionary analyses that have identified homologs of this network in several angiosperm lineages. ZAR1 is one of the most widely conserved NLRs in angiosperms and is evolutionarily ancient (Lewis et al., 2010). Its origins date back to the Jurassic period where it likely arose from a genome duplication event before the last common ancestor of the Eudicots and Monocots (Adachi et al., 2021; Gong et al., 2022). Additionally, the ZAR1 sequence is highly conserved including motifs that are essential for its resistosome function and RLCK binding (Adachi et al., 2021). Orthologs of *A. thaliana* ZRKs (AtZRKs) also share a similar distribution pattern to AtZAR1 orthologs and have been identified across several angiosperm lineages (Gong et al., 2022). Moreover, the ZRK family size varies widely across species, which may reflect varying roles of the RLCK network in pathogen detection (Gong et al., 2022). These evolutionary analyses suggest that the loss of an AtZAR1 ortholog in a lineage was followed by loss of the corresponding ZRKs (Gong et al., 2022). Furthermore, AtZAR1 orthologs are under strong negative selection at the AtZAR1-AtZRK1/RKS1 interaction interface suggesting that ZAR1 interacts with ZRK proteins across angiosperm lineages (Gong et al., 2022). Indeed, co-immunoprecipitation (co-IP) experiments have shown that AtZAR1 orthologs in various plant species such as *Solanum lycopersicum* (tomato), the *Magnoliid Liriodendron chinense* (Chinese tulip tree), and the monocot *Colocasia escuelenta* (taro), among others are able to interact with their corresponding ZRKs (Gong et al., 2022). PBLs have also been identified across angiosperm species but their ability to interact with ZAR1 and ZRKs in non-*A. thaliana* plant species remains to be investigated (Bastedo et al., 2019; Gong et al., 2022).

In *Nicotiana benthamiana*, NbZAR1 interacts with XOPJ4 IMMUNITY 2 (JIM2), a member of the RLCK XII family of proteins and a paralog of ZED1, to mount an immune response against the *Xanthomonas perforans* YopJ family effector XopJ4 (Schultink et al., 2019). Like the ZRKs, JIM2 lacks a conserved motif thought to be essential for kinase activity and is therefore hypothesized to be a pseudokinase. However, JIM2 has not been shown to interact directly with XopJ4 and therefore it remains to be determined whether JIM2 and NbZAR1 are sufficient for the recognition of XopJ4 or if there are additional components involved in this effector recognition response (Schultink et al., 2019). The ZAR1-ZED1 recognized effector HopZ1a has been shown to be recognized in not only *A. thaliana* but also soybean (*Glycine max*), sesame (*Sesamum indicum*), rice (*Oryza sativa*), false flax (*Camelina sativa*), canola (*Brassica napus*), and *N. benthamiana* (Ma et al., 2006; Baudin et al., 2017; Breit-McNally et al., 2022). As NbZAR1 can associate with AtZED1 to recognize HopZ1a in *N. benthamiana* (Baudin et al., 2017; Schultink et al., 2019), it is plausible that this recognition of HopZ1a might occur in other plant species through their respective ZAR1 orthologs as well.

## Conclusion

Our understanding of NLR activation has benefited immensely over the past decade from the many functional studies into ZAR1, its kinase sensors, and its cognate effectors, including the first structure of a plant resistosome. The earliest framework for ZAR1 activity – recognizing a single effector, HopZ1a, within the “gene-for-gene” resistance framework (Kaloshian, 2004; Lewis et al., 2010) – has blossomed into an elaborate network of interactions between the ZRK and PBL kinase families, enabling ZAR1 to broadly recognize at least six effector families from two phytopathogenic bacterial species. Whether such a dynamic ZAR1-associated network exists beyond *A. thaliana* remains to be determined, but the identification of JIM2 in *N. benthamiana* and the conservation of key residues involved in RLCK interactions and ZAR1 oligomerization in flowering plants strongly suggests that it will be (Adachi et al., 2021; Gong et al., 2022). It will also be exciting to see whether the ZAR1 immune network can use kinases beyond the ZRKs and PBLs and whether it can trap effectors from non-bacterial phytopathogens, particularly given that both fungal and oomycete effectors can also target cytoplasmic kinases (Irieda et al., 2019; Liang et al., 2021).

As outlined in **Figure 1A**, the most apt parallel in plant immunity for ZAR1 is the small group of hNLR gene families, such as ACTIVATED DISEASE RESISTANCE 1 (ADR1) and N REQUIREMENT GENE 1 (NRG1) in *A. thaliana*. Much like ZAR1, the ADR1 and NRG1 families are also indispensable for multiple effector recognition events (Jubic et al., 2019), are activated to become calcium channels that can directly induce cell death (Jacob et al., 2021), and are reliant on numerous intermediary effector ‘sensors’. Unlike ZAR1, however, the hNLRs rely on a network of sNLRs instead of a kinase sensor network to recognize effectors. Overall, both ZAR1 and hNLRs rely on a network of effector sensors to confer broad spectrum effector recognition; however, we propose that the ZAR1/kinase network is likely to have been evolutionarily tailored to broadly monitor effector-induced kinase PTMs and the switch-like changes they produce.

## Author contributions

CB-M, BL, RAS, DD and DSG wrote the manuscript. All authors contributed to the article and approved the submitted version.

## Funding

This work is supported by Natural Sciences and Engineering Research Council of Canada Discovery Grants (DSG and DD), Natural Sciences and Engineering Research Council of Canada Postgraduate Awards (CB-M and BL), an Ontario Graduate Scholarship (CB-M), and the Centre for the Analysis of Genome Evolution and Function (DSG and DD).

## Conflict of interest

The authors declare that the research was conducted in the absence of any commercial or financial relationships that could be construed as a potential conflict of interest.

## Publisher’s note

All claims expressed in this article are solely those of the authors and do not necessarily represent those of their affiliated organizations, or those of the publisher, the editors and the reviewers. Any product that may be evaluated in this article, or claim that may be made by its manufacturer, is not guaranteed or endorsed by the publisher.
